# Supplementary material for: Development of an interactive web dashboard to facilitate the reexamination of pathology reports for instances of underbilling of CPT codes
Source: J Pathol Inform. 2023 Jan 12;14:100187. doi: 10.1016/j.jpi.2023.100187 (PMC9867971; doi:10.1016/j.jpi.2023.100187)
Supplement: Supplementary file 1 — Supplementary material [file mmc1.docx]

**Supplementary:**

**Supplementary Methods**

*Toggling different SHAP interpretations for CPT code models.* This dashboard works primarily with multi-class or multi-target classification of clinical text. Users can toggle between displays that predict the output of *ancillary codes* (i.e., separate models predicting binary endpoints to identify 38 CPT codes, each given a probability 0-1, multiple codes predicted simultaneously) and *primary codes* (i.e., one model to predict presence of one CPT code from a set made up of codes 88302, 88304, 88305, 88307, and 88309). For the *ancillary code* model, the application can only display the SHAP values for one CPT code at a time, so we included a selectable plot of each model’s predictions across the codes in order to quickly switch between the CPT code-specific models. The *primary code* model that we developed also has unique SHAP values for each potential code, so the scatter plot is also used to switch between codes to display correspondent text identified by the model.

*Filtering documents to focus on false positive reports as candidate underbilling instances.* In prediction mode, certain filters can then be applied to select the type of report that the dashboard will present. It is through these filters that we can restrict the dashboard to display false positives only; this is done by selecting the “False predictions” and “Positive only” filters (**Figure 2C**). This filter will show the reports that the model predicts to feature a certain code, but that the original coder did not assign. The coder can then select which codes it identifies from a dropdown menu. Pressing “Next report” updates a CSV file containing the coder's predictions. From there we can compare their predictions to both the original predictions and our model predictions.

*Focused search of keywords*. Even when not in the hands of coders, the dashboard has a search feature that makes it advantageous to work with. For example, if we believed that our model was particularly good (or perhaps particularly weak) at assigning codes pertaining to examination under a microscope to reports containing the word cytogenetics, then we easily analyze those further. To do this we would select the search by “Report” and search by “Code description” options, and search for “cytogenetics microscope” (**see Figure 2**). Clicking on any of the reports would then display that report on the main dash.

**Supplementary Results**


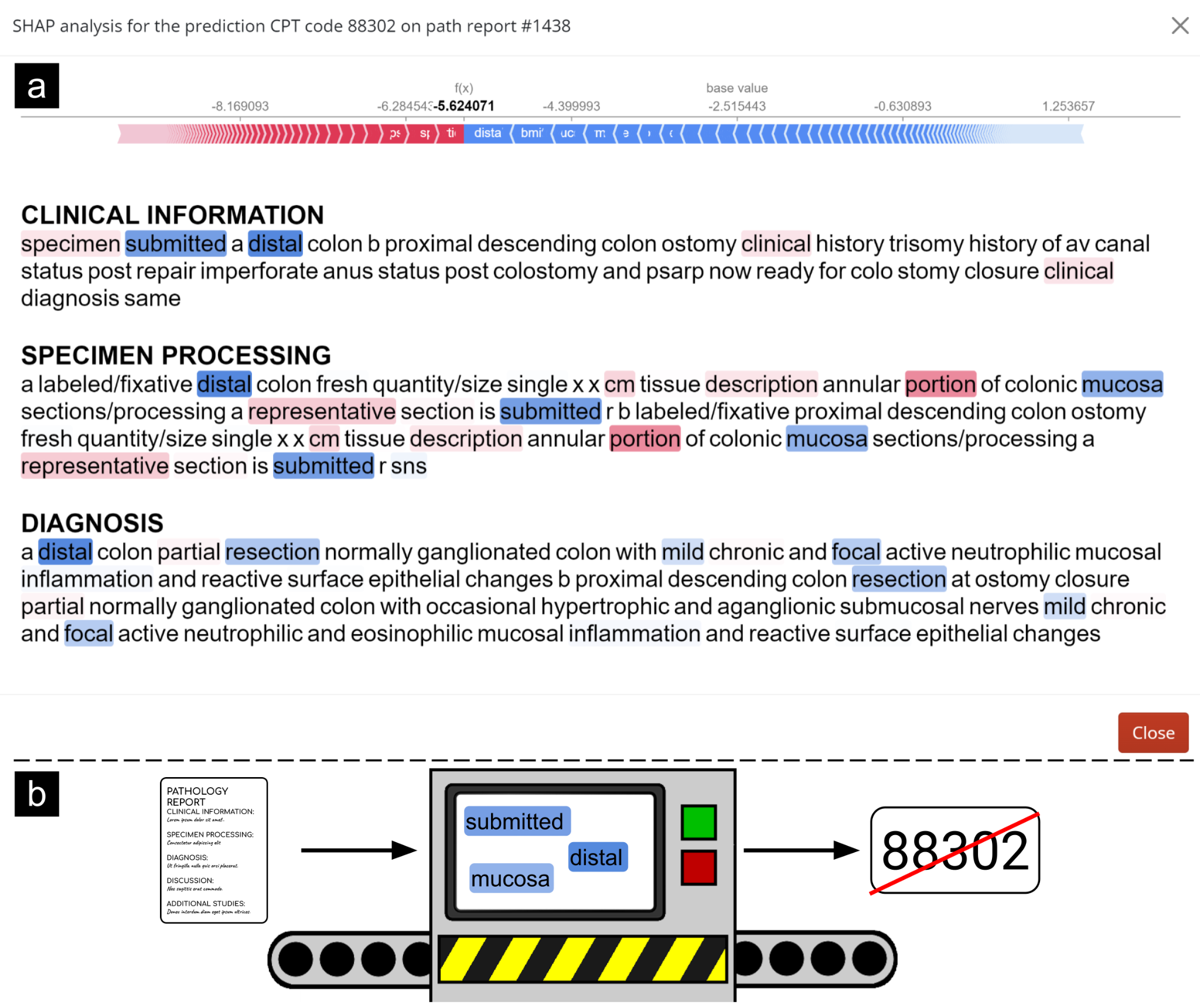


**Supplementary Figure 1:** Evaluation of same case from **Figure 3**, depicting words related to CPT 88302 using SHAP on #1438


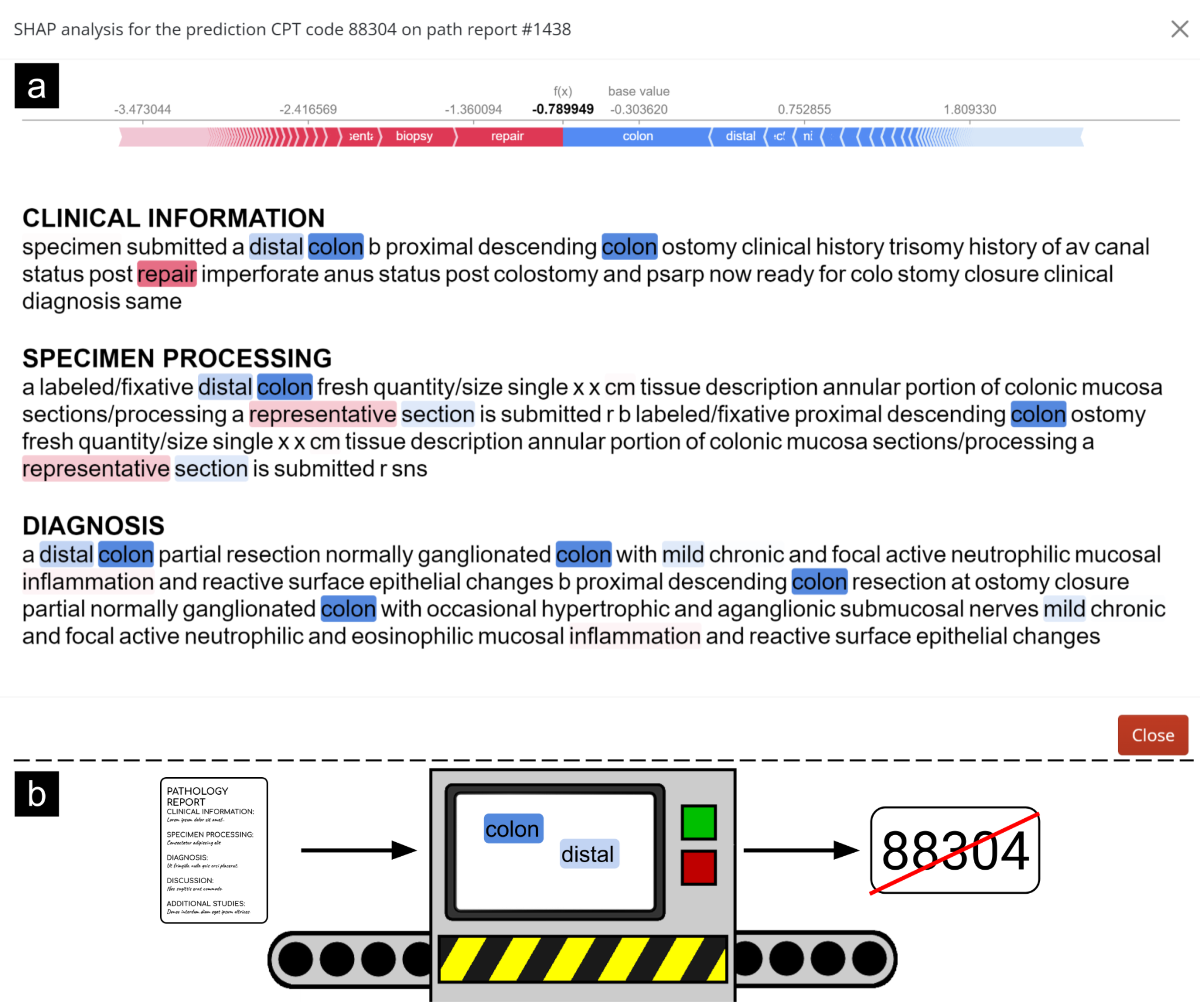


**Supplementary Figure 2:** Evaluation of same case from **Figure 3**, depicting words related to CPT 88304 using SHAP on #1438


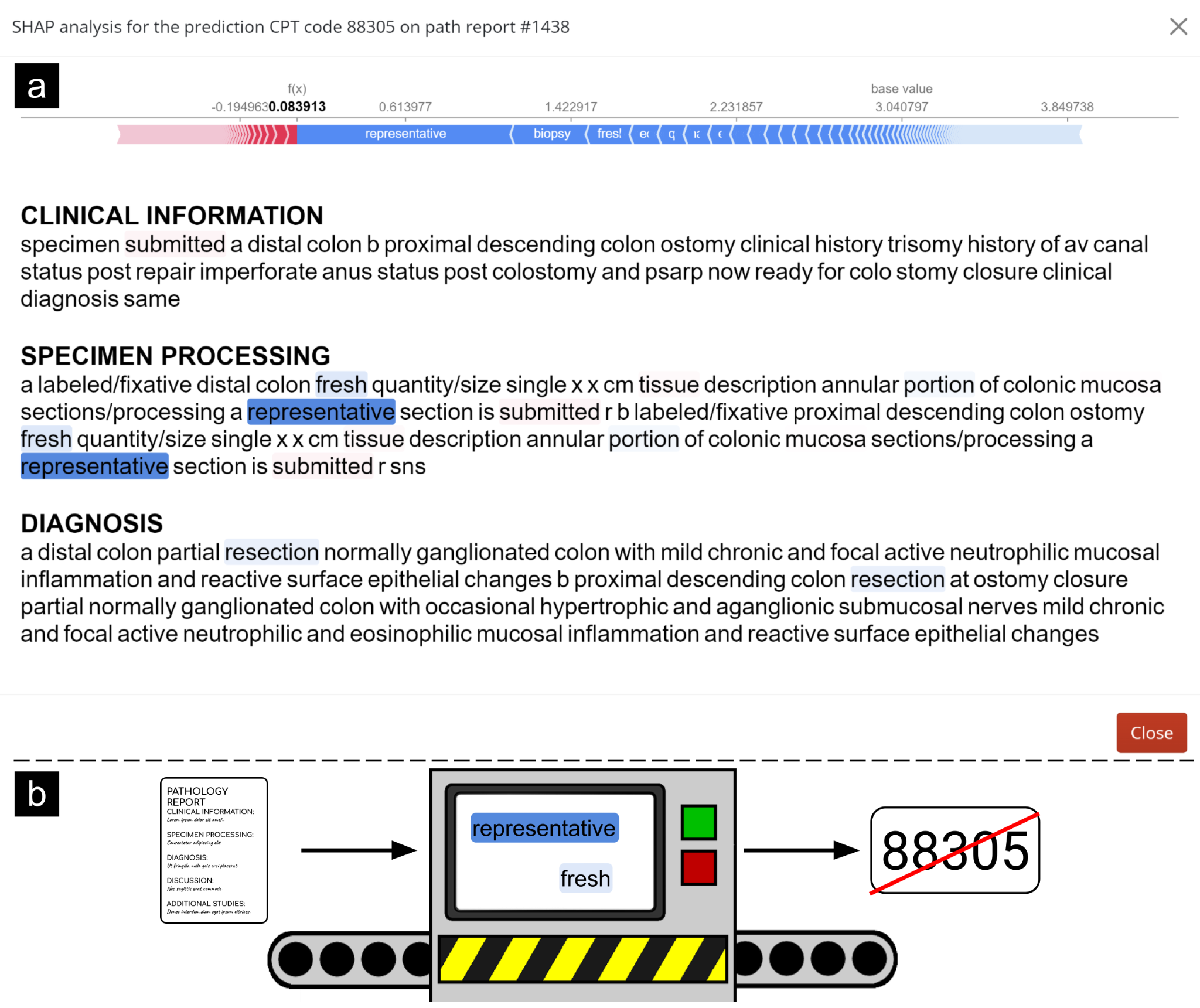


**Supplementary Figure 3:** Evaluation of same case from **Figure 3**, depicting words related to CPT 88305 using SHAP on #1438


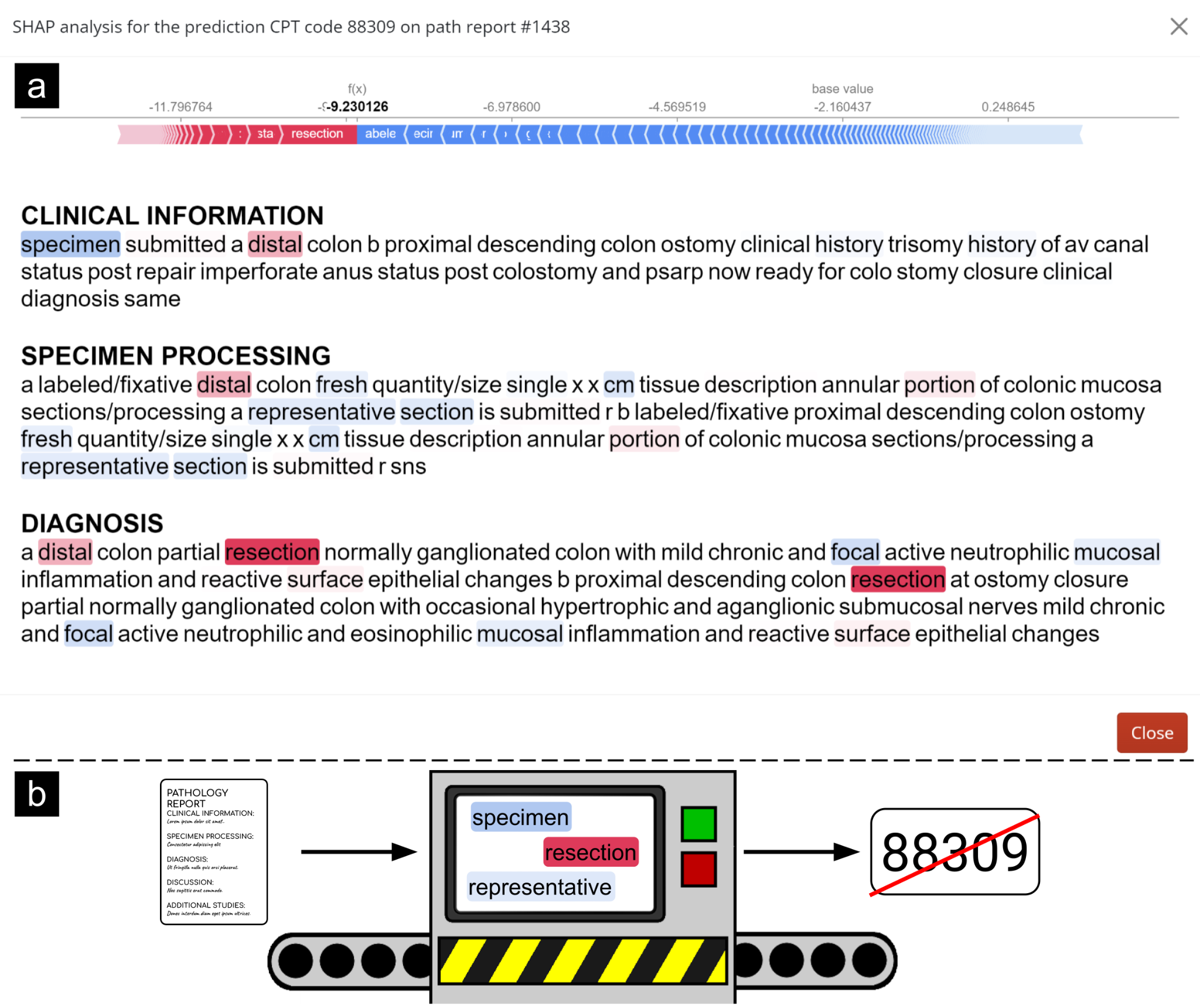


**Supplementary Figure 4:** Evaluation of same case from **Figure 3**, depicting words related to CPT 88309 using SHAP on #1438


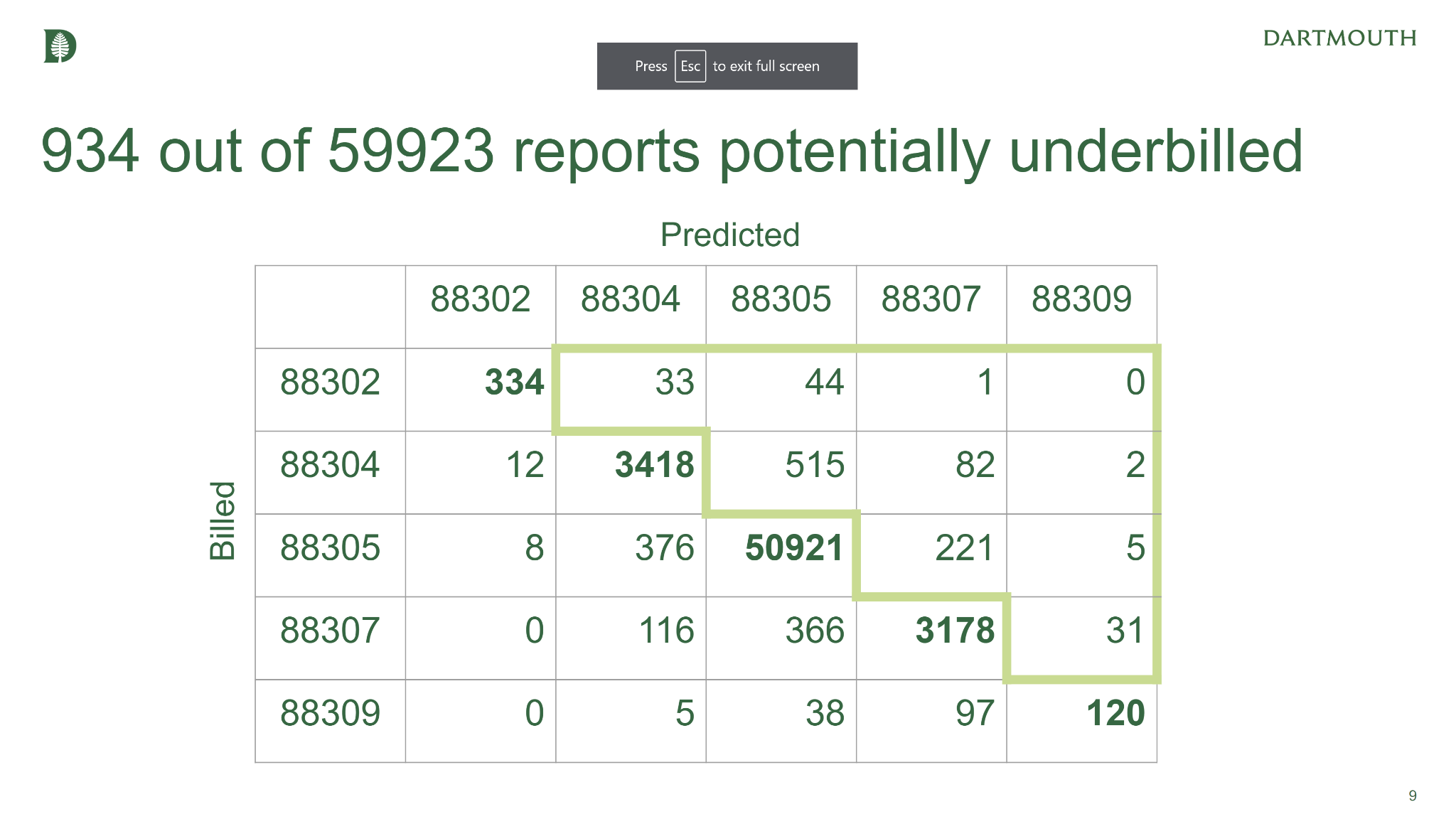


**Supplementary Figure 5:** Breakdown of all codes assigned a single primary code in our corpus. Candidate underbilled codes are highlighted in green


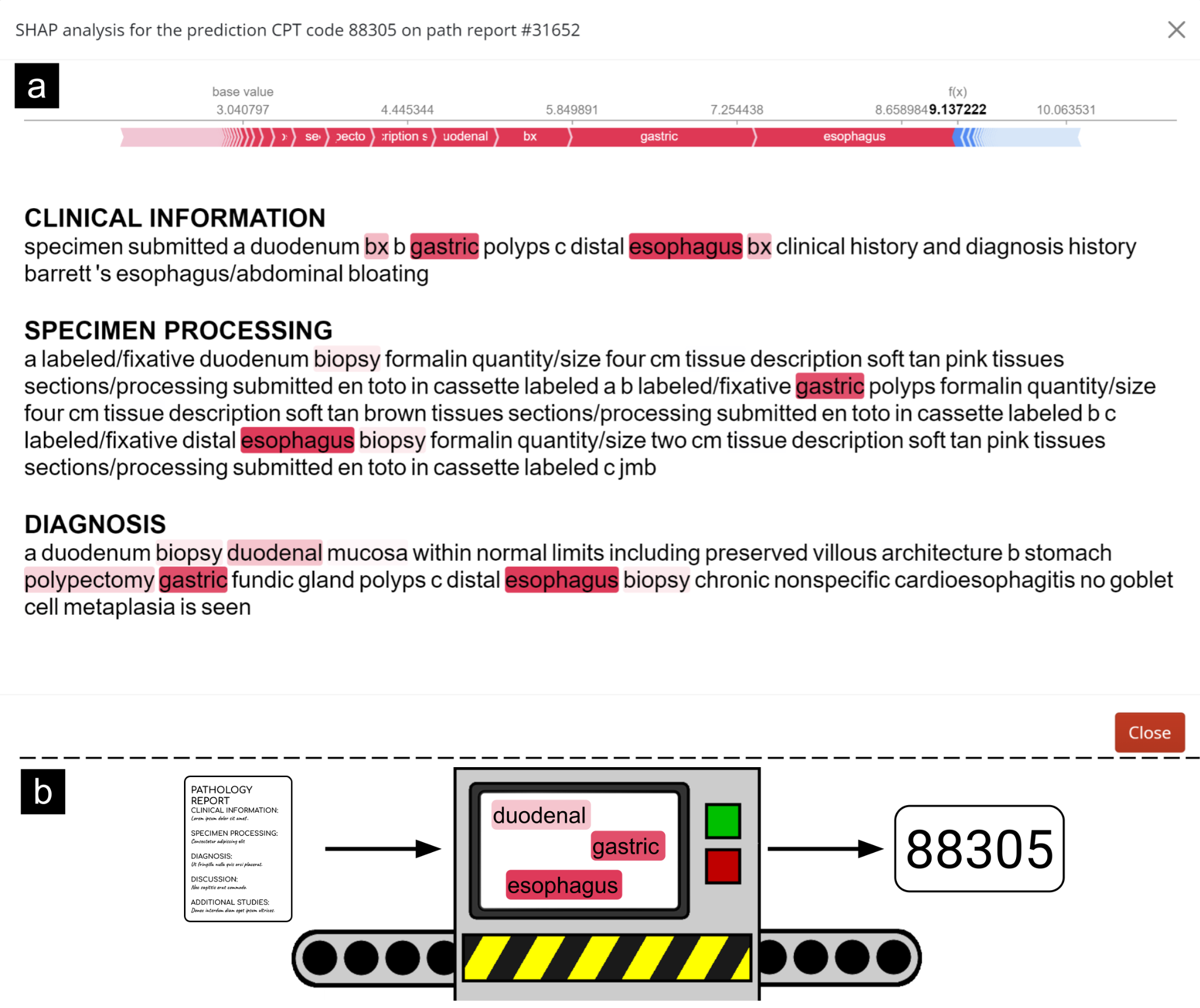


**Supplementary Figure 6:** Example interpretation plot for primary CPT code 88305. This report was correctly assigned CPT 88305 by both the coder and model.


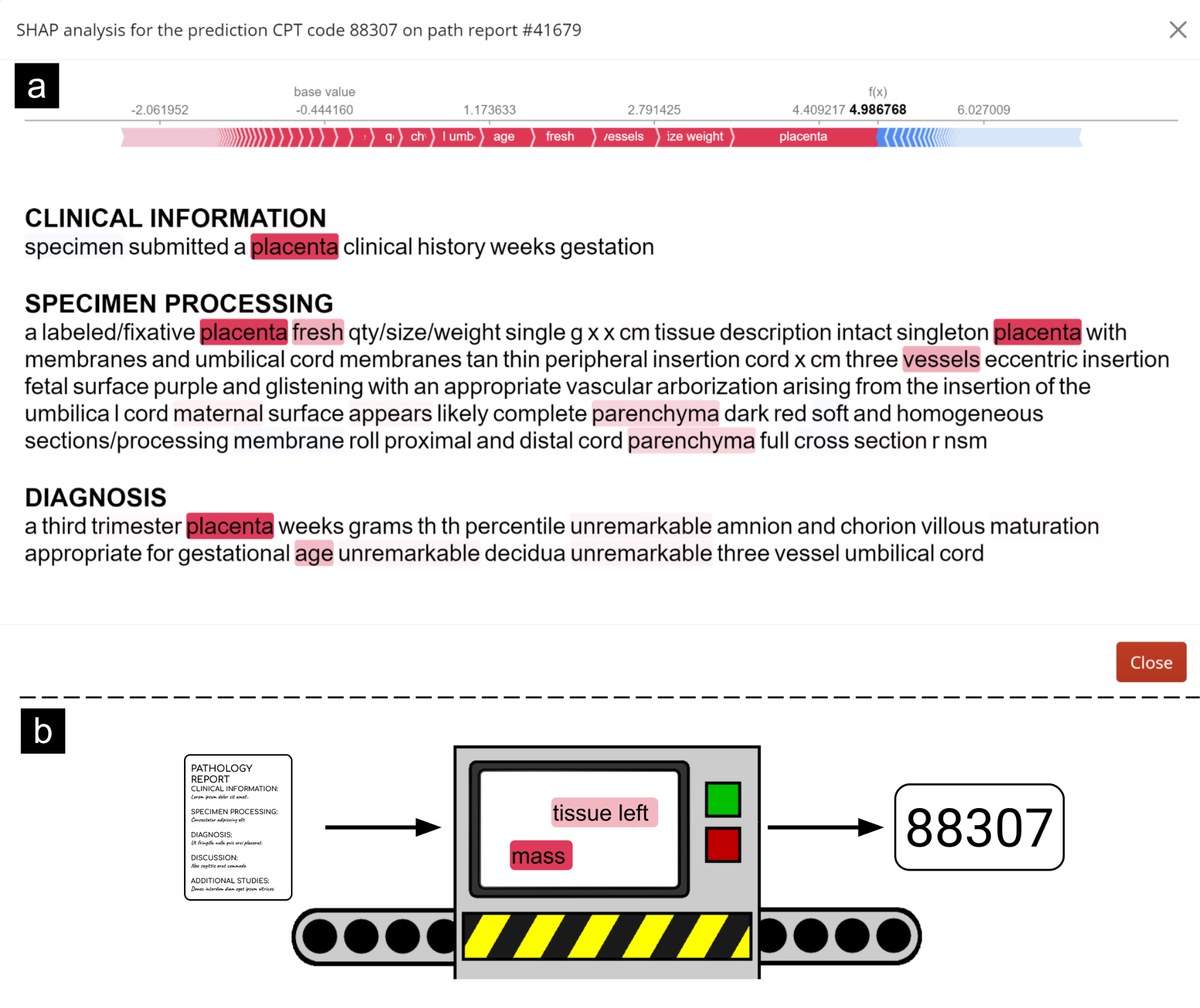


**Supplementary Figure 7:** Example interpretation plot for primary CPT code 88307. This report was correctly assigned CPT 88307 by both the coder and model.


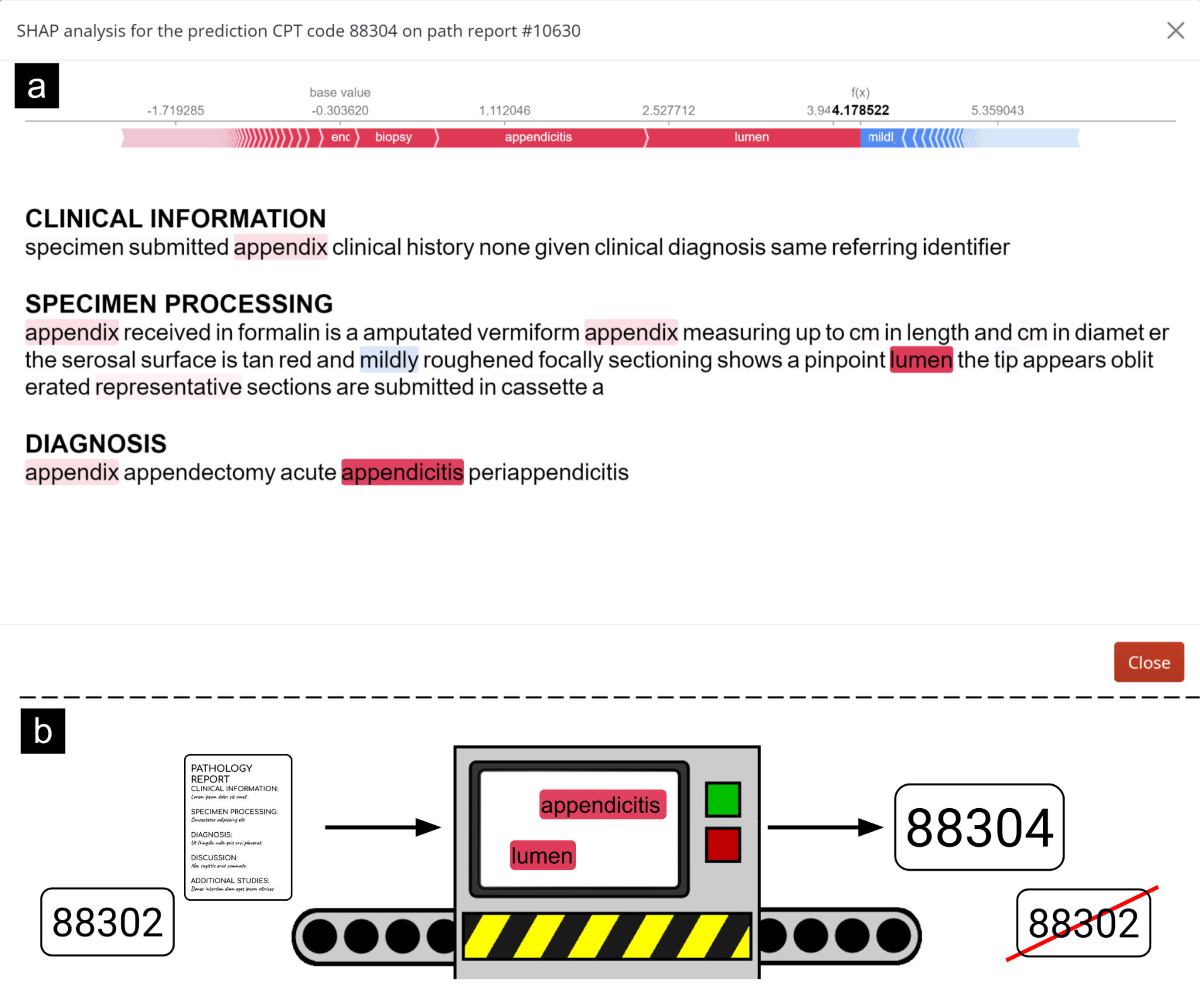


**Supplementary Figure 8:** Example interpretation plot for primary CPT code 88304. This report was assigned CPT 88302 by the coder, while the model predicted CPT 88304.


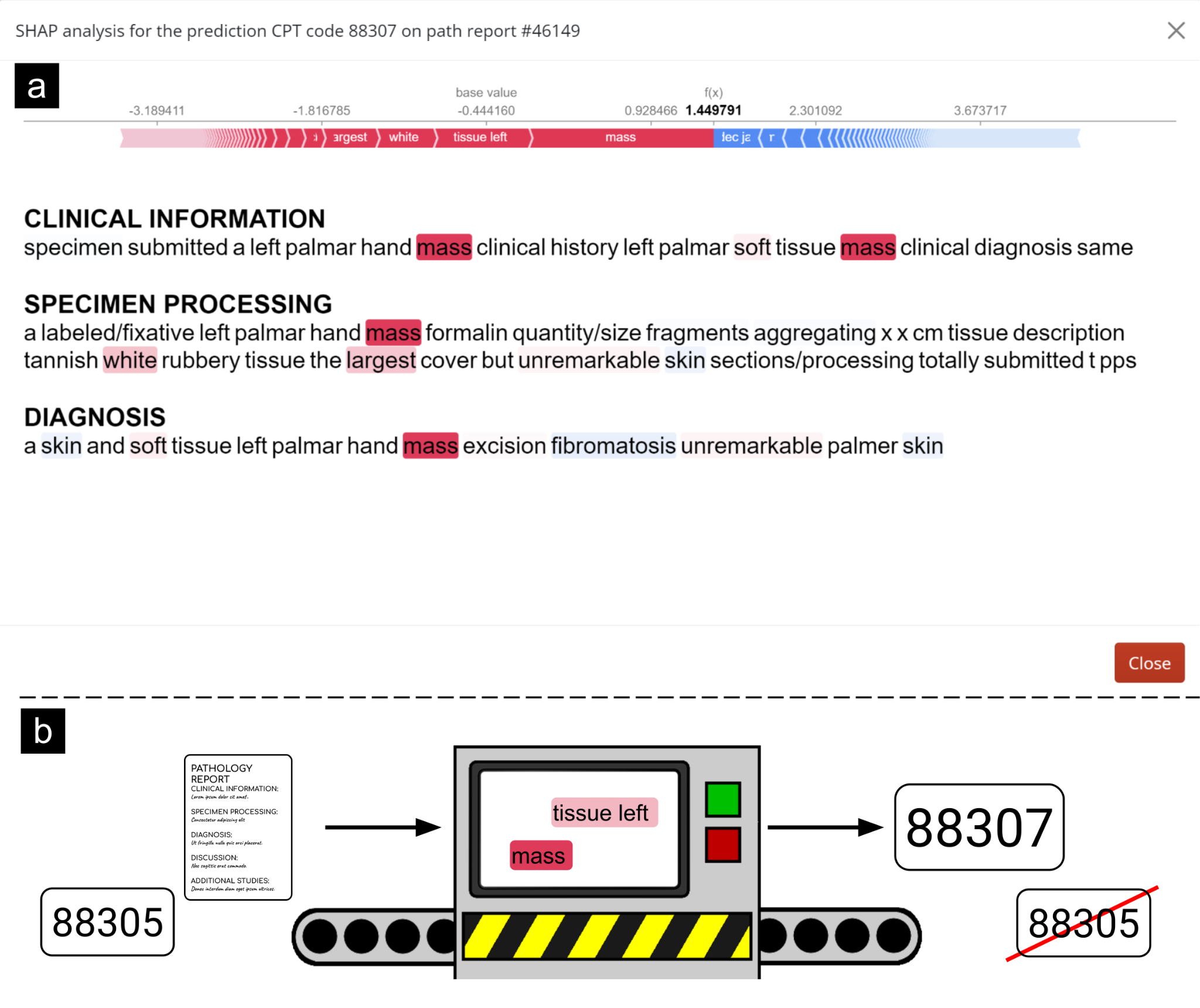


**Supplementary Figure 9:** Example interpretation plot for primary CPT code 88307. This report was assigned CPT 88305 by the coder, while the model predicted CPT 88307.
